# Tophaceous gout in a female premenopausal patient with an unexpected diagnosis of glycogen storage disease type Ia: a case report and literature review

**DOI:** 10.1007/s10067-016-3290-1

**Published:** 2016-05-02

**Authors:** Bingqing Zhang, Xuejun Zeng

**Affiliations:** 1 Department of Internal Medicine, Chinese Academy of Sciences and Peking Union Medical College, Peking Union Medical College Hospital, Beijing, China; 2 Department of General Internal Medicine, Chinese Academy of Sciences and Peking Union Medical College, Peking Union Medical College Hospital, No. 1 Shuaifuyuan Street, Dongcheng District, Beijing, China

**Keywords:** Glycogen storage disease type Ia, Gout, Hyperuricaemia

## Abstract

A young female with recurrent tophaceous gout and infertility presented to our clinic. On clinical evaluation, hypoglycaemia, hypertriglyceridaemia, lactic acidosis, and hepatomegaly were noted. Targeted gene sequencing revealed a novel composite heterozygous c.190G>T/c.508C>T mutation in the *G6PC* gene of the patient, leading to a diagnosis of glycogen storage disease type Ia. Her father possessed a heterozygous c.190G>T mutation, and her mother possessed a heterozygous c.508C>T mutation. A search of the previous literature revealed 16 reported cases of glycogen storage disease type Ia with gout. Here, we describe a female patient with gout, review previous cases, and discuss the mechanisms of gout and hyperuricaemia in glycogen storage disease type Ia.

## Introduction

Gout is a common chronic crystal arthritis predominantly found in elderly males. Gout in females, however, is less common, especially in premenopausal women; according to the UK database, the incidence of female gout with an onset age younger than 20 years is 1/10,000 [1]. In young female gout patients, genetic causes should be carefully evaluated. Glycogen storage diseases are a group of inherited disorders characterized by impaired glycogen utilization in the liver or muscle. Gout and hyperuricaemia are common presentations [2]. Here, we report the atypical case of a female premenopausal patient with a diagnosis of gout who possessed a novel composite heterozygous mutation of the *G6PC* gene, leading to a diagnosis of glycogen storage disease type Ia (GSD-Ia). We then reviewed the previous reports of GSD-Ia with documentation of gout.

### Case report

A 27-year-old female presented to the Gout Clinic of Peking Union Medical College Hospital with recurrent arthritis and infertility. Fourteen years before presentation, she noticed protrusion of her right ankle but did not undergo evaluation at a hospital. Eight years before presentation, at age 19, she experienced acute pain and swelling of her right ankle. The pain resolved spontaneously but recurred 6 to 7 times/year, involving the bilateral metatarsophalangeal (MTP) joints, bilateral ankles, and right knee. Her serum urate level (SUA) was elevated to 789 μmol/L. A diagnosis of gout was made, and she was prescribed NSAIDs for pain relief and allopurinol and benzbromarone to lower her urate level. However, she discontinued allopurinol and benzbromarone on her own as these medications triggered acute flares. Five years before presentation, she noticed multiple nodules in her MTP joints, ankles, and fingers. The nodules on her feet interfered with normal walking. She also complained of the inability to conceive after 3 years of attempts. Her age at menarche was 14 years, and her cycles were irregular. She was reported to prefer snacks and fatty food during her childhood. Her father had hypertension. Her grandfather had an SUA level greater than 400 μmol/L but without gout. On physical examination, the patient was generally healthy, with a height of 168 cm and a weight of 55 kg. Her blood pressure was 120/80 mmHg. Cardiac and pulmonary examinations were normal. On abdominal palpation, the liver was enlarged with a normal soft texture. A joint examination revealed multiple nodules on the bilateral MTP1 joints, ankles, and fingers.

A serological examination after overnight fasting revealed a SUA level of 548 μmol/L, serum creatinine (Cr) level of 49 μmol/L, fasting glucose level of 3.6 mmol/L, total triglyceride level of 6.22 mmol/L, and total cholesterol level of 6.60 mmol/L. Her resting lactate level was 7.4 mmol/L. Her 24-h urine urate level was 2.262 mmol/24 h, and her fractional excretion of uric acid (FE-UA) was 2.48 %. Her liver enzymes were normal. The estrogen level and basal body temperature curve were normal for her age and menstrual status. Renal ultrasonography revealed normal kidney size with possible calcium deposits in the bilateral renal medulla. Computed tomography (CT) imaging of her abdomen showed hepatomegaly without nodules. The pelvic ultrasonography and salpingography findings were normal. Serum examinations of her parents and her husband were normal.

Whole blood DNA was extracted from all four family members (the patient, both parents, and her husband) after signing an informed consent form. A genetic study was first conducted with a target gene sequencing approach. A search of the OMIM, NIH, and PubMed databases using the key words “FEMALE,” “GOUT,” and “HYPERLACTACIDEMIA” indicated familial juvenile hyperuricaemic nephropathy, glycogen storage disease type I, and glycogen storage disease type II. Next-generation sequencing was used to sequence each of the exons of the *UMOD*, *RENIN*, *G6PC*, *SLC37A4*, and *GAA* genes, as well as two SNP loci (rs2231142 and rs72552713) of *ABCG2* found in our previous report of a family including a female with gout.

**Fig. 1:**
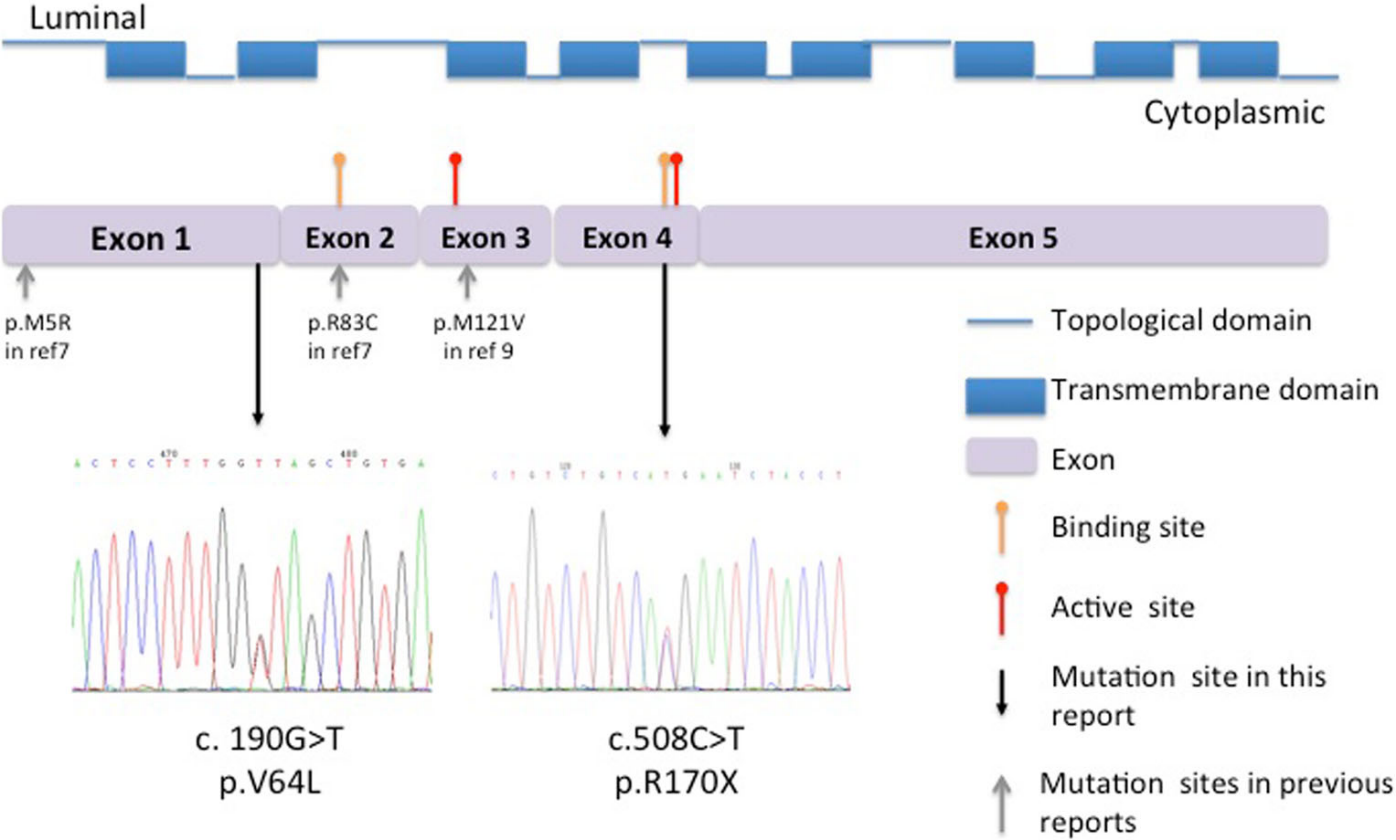
Protein structures of G6C and the mutations found in this study. The G6PC protein contains nine trans-membrane domains and ten topological domains. There are two binding sites on residue 83 and 170 (*yellow arrowhead*) and two active sites on residues 119 and 176 (*red arrowhead*). The two mutations, shown by the *black arrows*, were c.190G>T, resulting in p.V64L and c.508C>T, encoding p.R170X. The *gray arrowheads* indicate previous mutations reported in GSD1a patients complicated with gout

The genetic sequencing results showed that *UMOD*, *RENIN*, *SLC37A4*, *GAA* and rs72552713 of *ABCG2* were all normal in the patient and her family. Sequencing of the *G6PC* gene revealed composite heterozygous c.190G>T/c.508C>T mutations in the patient, a heterozygous c.190G>T mutation in her father, and a heterozygous c.508C>T mutation in her mother. The genetic analysis of her husband was normal. The c.190G>T mutation was located on exon 1 and encoded a missense mutation of p.V64L, whereas the c.508C>T mutation was located on exon 4 and encoded a nonsense mutation of p.R170X (Fig. 1). Sequencing of 50 healthy females and 100 healthy males at the two loci produced normal results. To our knowledge, the c.190G>T mutation has not been reported in previous literature.

### Literature review

We then searched PubMed using the keywords “GLYCOGEN STORAGE DISEASE TYPE IA” and “GOUT” and restricted the language to English. Patients with biopsy and/or gene sequencing confirmed GSD-Ia, as well as those with documented gouty arthritis, were reviewed, whereas those with other types of glycogen storage diseases and hyperuricaemia without gout flares were excluded. A total of 16 cases were available for review (Table 1). Among these cases, the male-to-female ratio was 9:7. Gout developed after puberty and was usually severe. Patient 1 had catastrophic axial gout resulting in irreversible paralysis. In contrast, the clinical presentations of glycogen storage were relatively mild, and ten (62.5 %) patients were not diagnosed until the gout was evaluated. All patients had hepatomegaly, and most exhibited hypoglycaemia, hypertriglyceridaemia, and lactate acidosis. Nine patients had recorded short stature, and four patients had recorded delayed puberty. Patient 4 experienced recorded irregular menses and a spontaneous abortion. Most of the patients were diagnosed by liver biopsy at a relatively late age (average age, 20.5 ± 3.06 years). G-6Pase enzyme activity was analyzed in five patients (patients 3, 4, 6, 8, and 11), which revealed partial or complete loss of enzyme activity. An additional four patients (patients 6, 7, 9, and 10) had undergone gene sequencing, two of which (patients 6 and 7) possessed a composite heterozygous p.R83C/p.M5R mutation, whereas the other two patients (patients 9 and 10) possessed a homozygous p.M121V mutation (Table 1).

**Table 1:** Summary of previous reports on gout in glycogen storage disease (GSD) type Ia

		Age of GSD (years)	Age of gout (yrs)	SUA (μmol/L)	Serum lactate (mmol/L)	Total triglyceride (mmol/L)	Fasting glucose (mmol/L)	Growth retardation	Reproduction system	Other presentation	GSD diagnosis	Ref
–^a^	F	27	19	789	7.4	6.22	3.6	No	Irregular menses, infertility		Sequencing^b^	
1	M	4	30	648	–^f^	2.37	–^f^	–^f^	–^f^	Anemia, paralysis due to axial gout	Liver biopsy	[3]
2	F	23	20	567	–^f^	13.08	3.1	–^f^	–^f^	Elevated liver enzyme, myopathy	Liver biopsy	[4]
3	M	18	21	492	2.08	–^f^		Yes	Delayed puberty	Renal failure; multiple hepatic cysts	Enzyme activity test	[5]
4	F	6	29	474	2.5	–^f^	3.6	Yes	Irregular menses, spontaneous abortion	Renal failure; multiple cysts	Enzyme activity test	[5]
5	M	28	16	830	10.2	2.14	2.9	–^f^	–^f^	Anemia; hepatic adenoma	Liver biopsy	[6]
6	M	17	17	680	7	3.26	3.9	–^f^	–^f^	Epilepsy	Liver biopsy, enzyme activity testing and sequencing^c^	[7]
7	F	14	14	625	4.42	3.30	–^f^	–^f^	–^f^		Sequencing^c^	[7]
8	M	0.75^e^	21	711	High	–^f^	Low	Yes	–^f^	Multiple liver adenomas	Liver biopsy, enzyme activity test	[8]
9	M	32	21	690	4.21	3.52	3.6	No	Normal	Multiple liver adenomas	Sequencing^d^	[9]
10	F	34	18	534	2.49	10.45	3.7	No	Normal		Sequencing^d^	[9]
11	F	40	14	792	3.54	21.89	4.3	Yes	Normal		Liver biopsy, enzyme activity testing	[10]
12	F	30	23	486	5.1	27.40	3.3	Yes	Normal	Decreased uric acid excretion	Liver biopsy	[11]
13	M	28	24	900	6.35	12.9	3.2	Yes	Normal	Decreased uric acid excretion	Liver biopsy	[11]
14	M	18	15	780	4.69	16.93	2.8	Yes	Decreased puberty		Liver biopsy of a sibling	[12]
15	F	32	24	590	12	Elevated	2.5	Yes	Delayed menarche		Intravenous galactose experiment	[13]
16	M	4	18	339	–^f^	17.5	2.8	Yes	–^f^		Liver biopsy	[14]

^a^The case presented in this report

^b^p.V64L/p.R170X composite heterozygotes

^c^p.R83C/p.M5R composite heterozygotes

^d^p.M121V homozygous

^e^Diagnosis of GSD at the age of 9 months

–^f^No records

## Discussion

This case of a female gout patient was unusual in several ways. The age of gout onset was early, and the disease course was severe, with formation of multiple large stones only 3 years after the first gout flare. She also exhibited hypoglycaemia, hypertriglyceridaemia, lactic acidosis, and hepatomegaly, indicating a metabolic abnormality. Irregular menses and infertility also raised suspicion. A genetic study revealed a novel composite heterozygous mutation of the *G6PC* gene. Given the typical clinical presentation and the presence of a composite heterozygous missense mutation, a diagnosis of GSD-Ia was made.

Gout and hyperuricaemia are common phenotypes observed in several inherited diseases [15]. GSD-Ia (OMIM: 232220), also known as Von Gierke disease, is a well-known autosomal recessive disease characterized by the inability to convert stored hepatic glycogen into circulating glucose [2]. Patients are usually diagnosed as babies, with a characteristic doll face, protruding abdomen and hepatomegaly, hypoglycaemic attacks, hypertriglyceridaemia, lactic acidosis, and growth and mental retardation. Patients usually fail to thrive if they are not properly treated [2].

Hyperuricaemia is also a feature, whereas gout is found only in those who reach puberty. Alepa et al. [11] reported decreased renal uric acid secretion in GSD-Ia patients (341∼381 mg/24 h; normal range 426 ± 81 mg/24 h). However, the finding that the uricosuric agent probenecid is ineffective to lower uric acid, whereas the xanthine oxidase inhibitor allopurinol can effectively maintain a normal SUA level, indicates that over-production might be the main mechanism of hyperuricaemia in GSD-Ia patients [12]. Jakovcic and Sorensen [12] used radioactive glycine-C14 to study the process of glycogen conversion in GSD-Ia patients. They found that the uric acid pool and daily production in GSD-Ia patients were 63 and 25 mg/kg, respectively, which are significantly higher than the normal values of 20 and 10 mg/kg, respectively; in contrast, the recovery of uric acid-C14 from the urine was 31.5 % of the injected dose, which was lower than the normal value of >65 %. Thus, in GSD-Ia patients, both over-production and under-secretion contributes to hyperuricaemia.

The culprit gene of GSD-Ia is *G6PC*, which encodes glucose-6-phophatase (G6Pase). Normally, G6Pase converts glucose-6-phosphate into glucose and inorganic phosphate (Pi), which is an important step to maintain normal blood glucose levels during glycolysis and gluconeogenesis. However, in the absence of G6Pase, glucose-6-phosphate accumulates in the cells and is then redirected into the pentose phosphate pathway, producing ribose-5-phosphate [12]. In the liver, ribose-5-phosphate degrades into phosphoribosyl pyrophosphate (PRPP), which is the first step in the uric acid de novo synthesis pathway [16]. In addition, the inability of glucose-6-phosphate to release Pi impairs the ATP compensation processes, resulting in an accumulation of the ATP degradation products ADP and AMP. ADP and AMP are then deaminated into IMP and enter the uric acid salvage synthesis pathway [17]. Thus, in GSD-Ia, both the de novo and salvage synthesis pathways of uric acid are accelerated. In addition, serum lactate has been shown to trans-stimulate the urate reabsorption transporters, urate transporter 1 (URAT1), and glucose transporter 9 (GLUT9) [18, 19], in the renal proximal tubules, resulting in increased uric acid reabsorption and decreased uric acid secretion.

The patient in this case and in four other cases reported irregular menses. In one study of 13 patients with GSD-Ia (mean age 11.2 years), all patients older than 4.8 years of age had a polycystic ovarian appearance and significantly higher basal and 2-h plasma insulin levels than the control subjects. The serum gonadotropin, androgen, IGF-I and sex hormone binding globulin (SHBG) levels were mostly normal [20]. In another study of 25 women with GSD-Ia, 8/25 (32 %) patients had delayed menarche (defined as ≥15 years of age), 12/25 (48 %) had irregular cycles, and 6/18 (33.3 %) had documented polycystic ovaries. In five patients with GSD-Ia, five successful spontaneous pregnancies and one aided pregnancy were reported [21]. The mechanisms of these abnormalities were unclear. Sechi et al. [21] found an association between age at GSD-Ia diagnosis and age at menarche (*p* = 0.0068) and between age at starting a corn starch-based diet and age at menarche (*p* = 0.01). They concluded that an early diagnosis with early implementation of a corn starch-based diet might prevent delayed puberty. The patient in this case had a normal ovarian appearance and female hormone levels but irregular cycles and infertility, which might be more complicated, involving other medical conditions, hormonal changes, and psychological and social factors.

*G6PC* contains five exons encompassing 12.5 kb on chromosome 17q21. At present, over 100 mutations have been reported. However, only a limited number of mutations account for the majority of GSD-Ia cases, among which, c.648G>T (p.L216L), a splicing mutation, and c.248G>A (p.R83H), a missense mutation, are the most prevalent mutations in the Chinese, Japanese, and Korean populations [22, 23], whereas c.247C>T (p.R83C) and c.1039C>T (p.Q347X) are the most prevalent mutations among Caucasians [23]. The G6Pase protein is a nine-trans-membrane protein with two binding sites (loci 83 and 170) and two active sites (loci 119 and 176) [22]. In the present case, c.190G>T encoded a missense mutation, p.V64L, in trans-membrane helix-2, whereas c.508C>T encoded a nonsense mutation of p.R170X in the second binding site (Fig. 1). The trans-membrane helices are crucial for correct folding, and abnormal proteins are designated for degradation [24]. The p.W63R/p.G68R mutation in helix-2 was shown to result in reduced levels of the G6Pase protein [24], whereas the p.M121V mutation in helix-3, found in patients 9 and 10, resulted in 7.8 % of normal activity during an in vitro expression study [9]. The binding sites (loci 83 and loci 170) and active sites (loci 119 and 176) compose the catalytic center, and mutations at these sites, namely p.R83C, p.R83H, p.H119L, p.R170Q, and p.H176A, completely abolish G6Pase enzymatic activity [24]. Carves et al. [7] reported a young male patient with no enzymatic activity in a liver biopsy specimen who was found to possess a heterozygous p.R83C/p.M5V mutation. Thus, although we did not test enzymatic function, the p.V64L/p.170X mutation could be assumed to impair normal protein function.

Of the five cases reviewed here with tested enzymatic activity, the patient (patient 11) with partial enzymatic function deficiency (2.40 ± 1.98 μmol Pi min^−1^ g^−1^, normal 4.7 ± 1.9 μmol Pi min^−1^ g^−1^) had a relatively mild manifestation of GSD-Ia, whereas the remaining four patients (patients 3, 4, 6, and 8) with complete enzymatic function deficiency presented with early and typical manifestations of GSD-Ia. Thus, the clinically mild GSD-Ia phenotype in the present case might be related to a partial enzymatic deficiency. However, one GSD-Ia patient with a homozygous p.P257L mutation, which revealed only 1.2 % of normal activity during an in vitro expression study, had a mild phenotype [25], whereas another patient with a composite heterozygous p.E110Q/p.G222R mutation, which retained 17 and 4 % of normal activity, respectively, manifested typical severe symptoms [26]. Variable phenotypes with the same G6PC genotype have been reported [27]. Thus, a genotype-phenotype correlation is lacking, and other modifying factors and/or genes might be present that affect the phenotype of GSD-Ia patients.

Frequent ingestion of uncooked corn starch is the main treatment for GSD-Ia and might improve growth and reduce mortality [28]. Potassium citrate is preferred to correct lactic acidosis [29]. Allopurinol, a xanthine oxidase inhibitor that blocks uric acid production, can effectively lower the SUA level, whereas uricosuric agents were not effective [12]. Liver transplantation was reported in one patient with gout (Patient 8). However, that patient’s SUA level increased from 484 to 711 μmol/L after transplantation, and he suffered from gout flares even after other serological parameters returned to normal [8]. Few reports exist on the treatment of GSD-Ia-related menstruation abnormalities, but early dietary intervention might be associated with correction of menstruation [21]. The patient in this report was advised to ingest complex carbohydrates as her main diet and to have frequent snacks between meals. She was also administered allopurinol with low-dose colchicine for gout flare prophylaxis. The tophi on her feet were removed surgically to enable normal walking. At the time of completion of this manuscript, her SUA level was 360 μmol/L, and she was preparing for in vitro fertilization.

In conclusion, here, we report the case of one female premenopausal gout patient with multiple metabolic abnormalities and infertility, who was finally diagnosed with GSD-Ia caused by a novel composite heterozygous mutation, c.190G>T/c.508C>T, encoding a missense mutation (p.V64V) in one trans-membrane helix and a nonsense mutation (p.R170X) in a binding site. Glycogen storage disease should be considered in young female patients with gout and hyperuricemia.
